# T Wave Alternans And Ventricular Tachyarrhythmia Risk Stratification: A Review

**Published:** 2003-04-01

**Authors:** Masahiko Takagi, Junichi Yoshikawa

**Affiliations:** Department of Internal Medicine and Cardiology, Osaka City University Graduate School of Medicine, 1-4-3 Asahimachi, Abeno-ku, Osaka 5458585, Japan

**Keywords:** T wave alternans, sudden cardiac death, ventricular tachyarrhythmia

## Abstract

Sudden cardiac death (SCD) is one of the leading causes of mortality in industrialized countries. Thus, identifying patients at high risk of SCD is an important goal. T wave alternans (TWA) is a new method for identifying patients with lethal ventricular tachyarrhythmias, and is dependent on heart rate. The maximal predictive accuracy is achieved at heart rates between 100 and 120 bpm, so that TWA is usually measured during exercise, phamacological stress, or atrial pacing. It has been shown that TWA has high sensitivity and negative predictive value for predicting SCD after myocardial infarction and is also useful for predicting SCD in patients with nonischemic cardiomyopathy. Although the implantable cardioverter defibrillator (ICD) is now the primary therapy for preventing SCD, it is difficult to identify those patients who are susceptible to lethal ventricular tachyarrhythmias for primary prevention. In the prediction of SCD, TWA can be used as a screening test of appropriate patients for further electrophysiological examination and therapy.

Sudden cardiac death (SCD) is a leading cause of mortality and remains a major clinical challenge [1]. Concerning therapeutic modalities, tremendous progress has been made in the development of the implantable cardioverter defibrillator (ICD). However, progress in identifying patients at high risk of SCD has lagged behind. Large multicenter trials have shown that electrophysiologic study (EPS) may be useful in identifying patients who would benefit from ICD therapy [2,3]. Unfortunately, EPS is costly, invasive, and imperfect [4]. Several noninvasive markers of risk-stratification have been studied and compared with EPS. Left ventricular ejection fraction (LVEF), frequent ventricular premature complexes (VPC) on Holter recording, and ventricular late potentials (LP) are all sensitive, but of low specificity and positive predictive value (PPV) [5,6]. Measurement of heart rate variability, especially in combination with LVEF, VPC, and LP, has significantly improved risk prediction, but PPV remains low [6]. A screening procedure that is more sensitive and specific - with high PPV for identifying patients at high risk of developing ventricular tachyarrhythmias - is needed.

Recently, assessment of repolarization alternans (T wave alternans [TWA]) in the electrocardiogram (ECG) has been suggested as a predictor of susceptibility to lethal ventricular tachyarrhythmias [7,8]. Although overt TWA in the ECG is not common [9], digital signal processing techniques capable of detecting subtle degrees of TWA (microvolt TWA) have shown that TWA may represent an important marker of vulnerability to ventricular tachyarrhythmias. This review discusses the electrophysiologic mechanisms that link TWA to arrhythmogenicity and the recent clinical data for its prognostic efficacy in predicting lethal ventricular tachyarrhythmias.

## History of TWA

TWA was first described in 1908; it is the variation in vector and amplitude of the T wave that occurs on an every-other-beat basis [10]. In a review by Kalter in 1948, 5 patients were identified with macroscopic TWA - a frequency of 0.08% [11]. In humans, macroscopic TWA has been associated with increased vulnerability to ventricular tachyarrhythmias under several pathophysiologic conditions such as myocardial ischemia [12-14], vasospastic angina [15,16], marked electrolyte abnormalities [17,18], hypertrophic cardiomyopathy (HCM) [19], the long QT syndrome [20-22], and the Brugada syndrome [23,24]. Microscopic TWA was first reported in 1982 [25]. Thereafter, many studies have led to the development of the method for detecting microvolt TWA and to the establishment of a relationship between TWA and susceptibility to ventricular tachyarrhythmias, in humans [26,27].

## Pathophysiology of TWA

### Ionic currents and TWA

There is some evidence that TWA is linked to alternations in cellular calcium homeostasis, which significantly influence the action potential duration (APD) 1982 [28]. In the failing heart, electrical remodeling is a recurring feature that has been associated with an increased risk of SCD 1982 [29]. The arrhythmogenesis is due to the functional expression of proteins to control calcium homeostasis.

Potassium channels may also play an important role in ischemia-induced TWA. The different sensitivity of KATP channel activation during ischemia between epicardium and endocardium may be linked to TWA at the cellular level 1982 [30-32].

### Arrhythmogenesis and TWA

Currently, the hypothesis regarding the arrhythmogenic mechanisms associated with TWA is based on the concept that heterogeneous prolongation and increased dispersion of repolarization produce reentrant ventricular tachyarrhythmias 1982 [33]. The heterogeneity in dispersion of repolarization results in a 2:1 appearance on the surface ECG and provides conduction block in the areas with prolongation of repolarization, which fractionates the wavefront and facilitates reentry. Shimizu et al. 1982 [34] studied an experimental model of long QT syndrome utilizing an arterially perfused wedge of canine left ventricular wall. When the preparation was paced at a critical fast rate, there was pronounced alternation of APD of mid-myocardial (M) cells, resulting in a reversal of the transmural repolarization sequence leading TWA in the unipolar ECG . The alternation of APD of M cells observed under long QT conditions may exaggerate transmural dispersion of repolarization and develop torsade de pointes. Pastore et al. 1982 [35] investigated TWA in Langendorff-perfused guinea pig heart using mapping of epicardial APD during pacing. The critical pacing rate induced concordant TWA, which developed to discordant alternans of APD and increased susceptibility to ventricular tachyarrhythmias.

## Measurement of microvolt TWA

**Figure 1:**
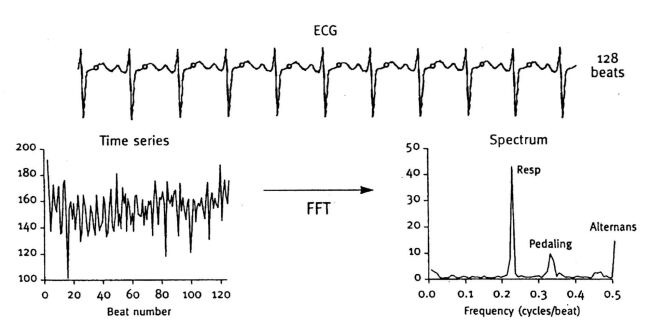
T wave alternans measurement: spectral method

Detection of microvolt TWA has been made possible by the use of advanced signal processing techniques and high-resolution electrodes to reduce noise. A number of beats, generally 128, are sampled and a time series of amplitudes of multiple corresponding points on the T wave are analyzed using a Fast Fourier Transform to generate a power spectrum (Figure 1). Several frequency peaks correspond to respiratory variation, pedaling (if bicycle exercise is performed), and noise. The presence of alternans is indicated by a frequency peak at 0.5 cycles per beat [36]. The analysis yields two measurements: the alternans magnitude and the alternans ratio. The former represents the magnitude of the alternating variation in T wave morphology compared to the mean T wave; a conventional threshold of 1.9 μV is used for significance. The latter is a measure of the statistical significance of the alternans with respect to the standard deviation of the background noise; it is generally required to be greater than 3, for significance. Furthermore, by definition, TWA must be sustained for more than 1 minute [37].

TWA is a rate-dependent phenomenon and microvolt TWA could develop in normal subjects at sufficiently high heart rate. It has been shown that the onset heart rate is relatively low in patients with structural heart disease and history of sustained ventricular tachyarrhythmia [38]. Kavesh et al. [39] showed that both TWA and false positive results increase with heart rate. Therefore, an onset heart rate of less than 110 beats per minute is a conventional requisite for positivity.

The original study of TWA was performed with atrial pacing to increase heart rate [40]. Either bicycle or treadmill exercise is now available with the use of high-resolution electrodes and advanced noise reduction algorithms. However, noise, premature beats, rapid changes in heart rate, or prominent beat-to-beat variability of RR intervals, may all mask true alternans [8]. All of these factors - plus the failure to achieve target heart rate - may result in an indeterminate test of TWA.

## Clinical studies of TWA for ventricular arrhythmic risk stratification

The first large clinical study of TWA was performed by Rosenbaum and co-workers [40], who observed 83 patients undergoing both EPS and TWA measured during atrial pacing. Over the following 20 months, ventricular tachyarrhythmic events occurred in 81% of patients with a significant level of TWA, compared with only 6% of those without. TWA was performed equivalently to EPS as a predictor of ventricular tachyarrhythmic events. Recently, Gold et al. [41] reported a prospective multicenter study of TWA, measured with bicycle exercise testing, in 313 patients referred for EPS because of syncope or presyncope, cardiac arrest, ventricular tachycardia, or supraventricular tachycardia. Signal-averaged electrocardiography (SAECG) was also performed at the time of TWA. Follow-up was obtained in 290 patients with a mean duration of 297 days. The predictive value of TWA and EPS for ventricular arrhythmic events was comparable, and better than SAECG . The combination of TWA with SAECG appeared to enhance the predictive value for ventricular arrhythmic events and the results of EPS.

### TWA in patients with prior myocardial infarction

Regarding the prognostic utility of TWA in patients with a prior myocardial infarction (MI), only a few studies have been reported. TWA, SAECG, and LVEF were measured in 102 patients with recent MI (20±6 days after the onset of the MI) [42]. A positive TWA test showed the highest sensitivity, relative risk, and negative predictive value (NPV) but also the lowest specificity, PPV, and predictive accuracy compared to SAECG and LVEF. With multivariate analysis, the combination of TWA and SAECG was the most significant predictor. Recently, a larger cohort study consisting of 836 patients who underwent TWA testing (2.7±5.4 months after the onset of the MI) revealed that TWA predicted SCD or resuscitated ventricular fibrillation (VF) [43].

The sensitivity, NPV, and risk hazard of TWA for predicting SCD or VF were higher than LVEF, SAECG, and the presence of non-sustained ventricular tachycardia (VT); however, the specificity and PPV remained worse. The utility of TWA for prognosis in patients with recent MI needs to be further clarified.

### TWA in patients with cardiomyopathy

Adachi et al. [44] reported a study of 58 patients with dilated cardiomyopathy (DCM) who underwent a TWA test. Analysis of recorded ventricular tachyarrhythmias, including non-sustained or sustained VT, revealed that ventricular tachyarrhythmias were more common in patients with a significant level of TWA (the sensitivity, specificity, and predictive accuracy rates of TWA to predict VT were 88%, 72%, and 77%, respectively).

Klingenheben et al. [45] studied 107 patients with congestive heart failure, a mean LVEF of 28±7%, and no history of sustained ventricular tachyarrhythmias. During 18 months of follow-up there were no patients in the TWA negative group that experienced an arrhythmic event or SCD. Multivariate Cox regression analysis revealed that TWA was the only independent predictor of arrhythmic events.

 A study of 104 patients with DCM, and with 12 arrhythmic events during 21±14 months demonstrated that TWA in a group of patients with an onset heart rate less than 100 beats per minute was the most significant predictor of arrhythmia-free survival (the sensitivity, specificity, PPV, NPV, and relative risk were 75%, 78.9%, 37.5%, 94.9%, and 7.4, respectively) [46].

Studies of TWA in patients with other cardiomyopathies are more scarce. Momiyama et al. [19] studied 14 patients with HCM. A significant level of TWA was found in 71% of 7 patients at high risk of ventricular tachyarrhythmias, compared with none of the other 7 patients who were at low risk. This result suggests that TWA may be a useful marker for high risk of ventricular tachyarrhythmias in patients with HCM; however, this finding was based on a small number of patients. The role of TWA for prognosis in patients with HCM needs to be elucidated.

### TWA in patients with the long QT and Brugada syndromes

Macroscopic TWA has been reported in patients with the long QT syndrome [20-22,34]. Prolongation and unstable state of the ventricular action potential may produce the macroscopic TWA and result in the polymorphic VT known as torsade de pointes. The prognostic value of microscopic TWA has not yet been assessed in patients with the long QT syndrome.

In patients with the Brugada syndrome, some reports have revealed that intravenous administration of class Ic antiarrhythmic drugs induced macroscopic TWA and resulted in VF [23,24]. These results suggest that in the Brugada syndrome class Ic antiarrhythmic drugs may accentuate the underlying sodium channel abnormalities, produce an unstable state of repolarization, increase the triggering PVC, and induce VF. On the other hand, Ikeda et al. [47] reported a low prognostic value of microscopic TWA in patients with the Brugada syndrome.

## Limitations of TWA

There are some technical and electrophysiologic limitations of TWA. The technical limitations are: 1) TWA cannot be measured in patients with atrial fibrillation, which is a common arrhythmia in patients with structural heart disease; and 2) the presence of frequent atrial or ventricular ectopy, excessive motion artifacts, and, in particular, the inability to achieve the target heart rate would render the results of the TWA test indeterminate. An incidence of indeterminate results of up to 25% is present in most of the published studies. The electrophysiologic limitation is that TWA testing may lose much of its predictive power within a few weeks after onset of MI.

## Perspectives

The clinical utility of TWA in evaluating arrhythmic risk stratification appears promising for patients with suggestive ventricular tachyarrhythmias, congestive heart failure or an LVEF of less than 40%, and a recent MI. The ultimate role of the TWA test as a noninvasive predictor of SCD awaits larger-scale prospective studies. In the near future, better definition of the clinical role of TWA test will be achieved with the results of the ongoing ABCD (Alternans Before Cardioverter Defibrillator) trial, in which ICD implantation will be performed in patients with ischemic cardiomyopathy and abnormal EPS and TWA results.
